# Surface-Enhanced Raman Spectroscopy on Self-Assembled Au Nanoparticles Arrays for Pesticides Residues Multiplex Detection under Complex Environment

**DOI:** 10.3390/nano9030426

**Published:** 2019-03-13

**Authors:** Yongmei Ma, Zhonghao Huang, Siyue Li, Chenghao Zhao

**Affiliations:** 1 Chongqing Institute of Green and Intelligent Technology, Chinese Academy of Sciences, Chongqing 400714, China; mayongmei@cigit.ac.cn; 2 Chongqing BOE of Optoelectronics Technology CO., LTD, No. 7 Yunhan Avenue, Shuitu Hi-Tech Industrial Zone, Chongqing 400714, China; lordhuangxixi@Outlook.com; 3 Department of Mechanical Engineering, City University of Hong Kong, Hong Kong 999077, China; chengzhao4-c@my.cityu.edu.hk

**Keywords:** self-assembly, SERS, pesticides residues, multiplex

## Abstract

The high reproducibility of trace detection in complex systems is very hard but crucial to analytical technology and science. Here, we present a surface-enhanced Raman scattering (SERS) platform made by large-scale self-assembly of Au nanoparticle (NP) arrays at the cyclohexane/water interface and its use for pesticides residues trace detection. The analyte molecules spontaneously localize into the Au NPs’ nanogaps during the self-assembly process, yielding excellent Raman signal enhancement by surface effects, and possibly both by the concentration of the analytes into the array and by plasmonic hot-spot formation. Transmission electron microscopy (TEM) images demonstrate a good uniformity of interparticle distances (2–3 nm) in the Au NP arrays. SERS experiments on crystal violet (CV) molecules demonstrated that the relative standard deviations (RSD) of the band intensities at 1173, 1376, and 1618 cm^−1^ were 6.3%, 6.4%, and 6.9%, respectively, indicating high reproducibility of the substrate. Furthermore, we demonstrate that two pesticides dissolved in organic and aqueous phases could be simultaneously detected, suggesting an excellent selectivity and universality of this method for multiplex detection. Our SERS platform opens vast possibilities for repeatability and sensitivity detection of targets in various complex fields.

## 1. Introduction

Molecular detection of target analytes such as pesticides [1], drugs [2], pharmaceutical molecules [3], biochemicals [4], neurotransmitters [5], and explosives [6] is urgently needed in many fields. Generally, real samples are mixtures that are commonly dispersed in a complex system, thus making identification and detection of target analytes a real challenge, especially for sensitive detection of analyte [7,8]. The problem is amplified when the signal of the analyte is swamped in the background signal of a complex system. The surface-enhanced Raman scattering (SERS) technique demonstrates a huge advantage with regard to this problem because of the high sensitivity and unique vibrational fingerprints [9,10,11]. SERS spectra with a narrow linewidth show promise in multiple detection under the complex fluids, including detection down to the single molecule level [12,13].

The enhancement of the Raman signal depends on the exciting localized surface plasmons within metallic substrates [14]. The design of a metallic substrate with a high density of hotspots can lead to a stronger signal, thereby lowering the limits of detection (LOD). Therefore, many studies make efforts to fabricate uniform and closely packed metallic nanostructures with multiple hotspots [15,16]. However, the optimal interparticle gaps, simple method of substrate preparation, and uniformity of the substrates are very important for sensitive SERS detection [17,18]. Meanwhile, the capacity of analytes to get into nanogaps decides the practicality and sensitivity of the substrates. Whereas, the protecting and capping agents in the precise nanofabrication will prevent analytes from reaching the nanogaps of the substrates and decrease the SERS sensitivity. Furthermore, precise nanofabrication is costly, non-scalable, and hard to clean.

Compared to traditional substrates, a self-assembled SERS platform is highly advantageous for practical applications because it can capture analytes into hotspots and control nanogaps easily [19]. The interfacial tension of different phases can be reduced by the adsorption of nanoparticles (NPs), which can stabilize the assembly [20,21]. A self-assembly strategy is an effective and low-cost method to fabricate an excellent substrate for trace SERS detection. A self-assembled nanostructure of SERS platform can create a multitude of hotspots and capture the analyte molecules dissolved in either the aqueous or the oil phases into hotspots during the self-assembly process. The two characteristics mentioned above can improve the SERS performance, which further exhibits the superiority of the interfacial self-assembled SERS platform. Our previous study has investigated drugs multiplex detection in urine using this self-assembled SERS platform and obtained good results [22]. Further investigation needs to be done for the advantages of this SERS platform in detecting other analytes, such as pesticides residues.

In this study, pesticides residues such as paraoxon and thiram were selected as model compounds for self-assembly SERS platform detection. Pesticides residues have aroused public concern on healthiness, environment, and food and water safety [23,24,25]. Ultrasensitive and accurate detection of pesticides residue levels in the surroundings is both necessary and beneficial to human health. However, the traditional methods, including gas/liquid chromatography combined with mass spectroscopy [26] and enzyme-based biosensors [27], suffer from high cost and intricacy. Consequently, a sensitive, simple, and cost-effective method for pesticides residues detection is still a challenge.

Herein, an interfacial SERS platform self-assembled from Au NPs arrays in cyclohexane (CYH)/water interface with a large scale was generated for trace pesticides residue detection. The analyte can be captured into nanogaps during the self-assembly processes, which ensure the high sensitivity of the interfacial SERS platform. Meanwhile, the large-scale substrate with ordered hotspots was beneficial to obtain high repeatable SERS signals. More importantly, no matter if they are hydrophilic, hydrophobic, or amphiphilic molecules, they can be captured into nanogaps from different liquid phases, or even an immiscible phase, so that they can be detected individually or simultaneously, which provides superiority for analytes detection under complex environments. Finally, a self-assembled SERS platform is easy to handle, self-healing, and does not need any additional assisted materials.

## 2. Materials and Methods

### 2.1. Chemicals and Materials

Chemicals including hydrogen tetrachloroaurate (HAuCl_4_·3H_2_O), sodium citrate, crystal violet (CV) and cyclohexane (CYH) were obtained from Shanghai Reagent Company, shanghai, China. 4-mercaptopyridine (4Mpy), thiram, paraoxon were obtained from Sigma Company (San Francisco, CA, USA). The solutions were prepared with ultrapure water without further purification and pH regulation, all experiments were conducted under room temperature 25 °C.

### 2.2. Apparatus

The morphological images were performed on a Sirion 200 field-emission scanning electron microscope (SEM) (ThermoFisher, Waltham, MA, USA) and JEOL 2010 high-resolution transmission electron microscope (TEM) (Japan Electronics Co., Ltd, Japan). The ultraviolet visible (UV−vis) spectrum was performed on a SHIMADZU-UV-2550 spectrophotometer (Shimadzu, Kyoto, Japan). Raman spectra were carried out on a LabRAM HR800 confocal microscope Raman system (Horiba Jobin Yvon, Longjumeau, France), using a 633-nm laser excitation source. The laser power was adjusted to approximately 0.5 mW. The laser was focused by a 50/0.5 NA objective lens. All the spectra reported were the results of a single 2 s accumulation.

### 2.3. Synthesis of Au NPs

Au NPs were prepared using a classical method [28]. A mixed solution consisting of 1 mL 1% HAuCl_4_·3H_2_O and 99 mL ultra-pure water was placed into round-bottomed flask with a reflux condenser, then brought to a boil with vigorous stirring. After that, 10 g/L trisodium citrate (2 mL) was added rapidly to the solution, maintaining the temperature of the mixed solution for 0.5 h. Finally, the obtained products were centrifugally precipitated and rinsed with ultrapure water.

### 2.4. Self-Assembly of Au NPs at Cyclohexane/Water Interface

Self-assembly of Au NPs was prepared according to the previously reported protocol [19,22]. A total of 10 mL of Au NPs aqueous colloid was collected by centrifugation (8000 rpm, 10 min). Subsequently, Au NPs were dispersed in paraoxon solution to a total volume of 2 mL in a watch glass with proper volume. Then, 200 μL cyclohexane was slowly added to the solution to form an immiscible liquid–liquid interface. Then, 1 mL of ethanol was rapidly injected into the mixed solution to entrap the Au NPs and analytes at the cyclohexane/water interface. After that, cyclohexane was evaporated spontaneously and the trapped Au NPs were simultaneously self-assembled into a large-scale highly close-packed array at the cyclohexane/water interface.

### 2.5. Surface Treatment of Silicon Wafer for Transferring the Self-Assembly SERS Platform

The silicon wafers were cleaned and hydroxylated by immersing in a mixture solution of 98% H_2_SO_4_ and 30% H_2_O_2_, 3:1, *v*/*v* for 1 h, followed by extensive rinsing with ultrapure water and drying under nitrogen. The silicon wafer has been used to transfer the interfacial SERS platform according to the previously reported protocol [19,29]. Briefly, the self-assembled Au NPs arrays can be easily transferred to a silicon wafer by bringing the silicon wafer parallel to the water surface and lightly touching the silicon wafer to the self-assembled Au NPs arrays. The self-assembled Au NPs arrays can be transferred to a Cu grid by the same method. Then, the silicon wafer and Cu grid with Au NPs arrays were used for SEM and TEM analysis, respectively. The results of SEM and TEM were used to estimate the diameter and gap of Au NPs.

## 3. Results and Discussion

### 3.1. Self-Assembled Au NPs Arrays at Cyclohexane/Water Interface

**Scheme 1:**
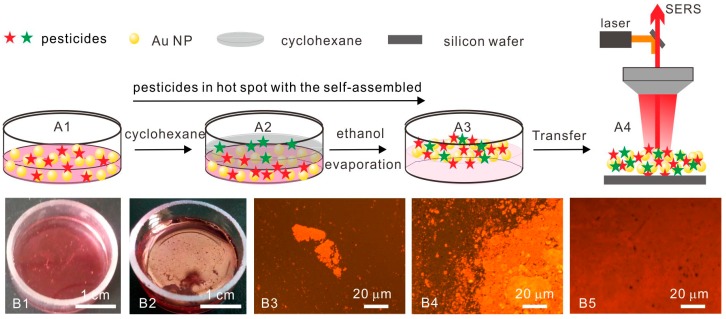
Schematic illustrations (**A1**–**A4**) and optical images (**B1**,**B2**) of self-assembly of Au NP arrays at the liquid interface for SERS platform and microscope images (**B3**–**B5**) of self-assembly process of the large-scale interfacial Au NP arrays.

Au NPs were synthesized according to the citrate reduction method. Then, interfacial Au NP arrays were prepared by self-assembled Au NPs at the immiscible cyclohexane/water interface, see Scheme 1. The analytes were directly captured into the interparticle nanogaps during the array formation process. Briefly, Au colloidal and pesticide mixed solutions were poured into a glass container, see Scheme 1A1,B1, cyclohexane was poured on top of the mixed solution, see Scheme 1A2, Au NPs were subsequently forced to assemble at the cyclohexane/water interface after the rapid addition of ethanol to the colloidal solution. After evaporation of the cyclohexane, the uniform and highly close-packed Au NP arrays was spontaneously formed over a large area by the principle of the total interface energy minimum, see Scheme 1A3,B2 [30,31].

The self-assembly process can cause a disordered nanostructure to form a long-range ordered structure because of the specific interactions among the components themselves, without external direction. The suitable balance of various forces is a key factor during the self-assembly process at the immiscible liquid interface, which ensures Au NPs form a close-packed array at the liquid interface without aggregation and precipitation [32]. The time-course figures clearly showed the whole process; meanwhile, the scale change of Au NP arrays from micrometer to centimeter levels were also displayed, see Scheme 1B3–B5. The collective plasmon resonance in Au NP arrays induced a golden color in reflection of Au NP arrays, see Scheme 1B2–B5 [33]. This large-scale self-assembly technology is easy to handle and thus makes it possible to possess practicability and the value of popularization.

**Figure 1:**
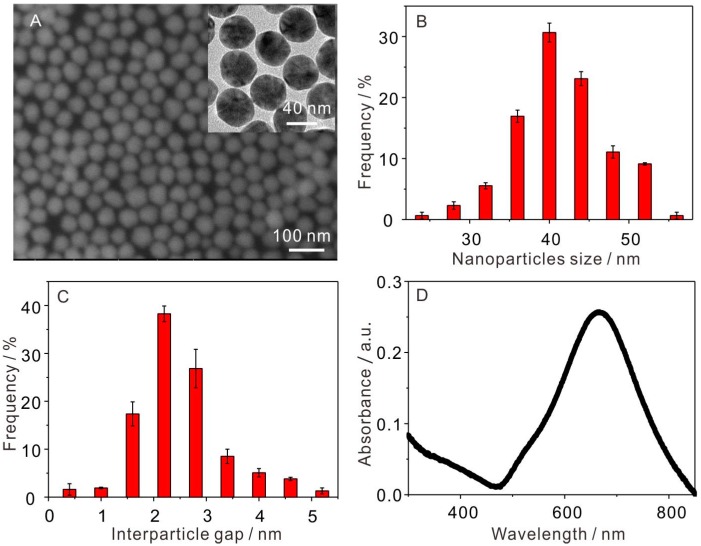
(**A**) SEM observations on Au nanoparticle (NP) arrays (inset in A: TEM of self-assembled Au NP arrays); (**B**) nanoparticle size distribution histograms of Au NPs arrays; (**C**) interparticle gap distribution histograms of Au NPs arrays; (**D**) absorbance spectra of self-assembled Au NPs arrays transferred onto a quartz glass surface.

The interfacial self-assembly Au NP arrays can be directly transferred onto quartz glass or Si wafer by placing them parallel to the interface, mildly touching and then pulling it slowly from the liquid interface. The geometric morphologies and structures of the interfacial self-assembly Au NP arrays were clearly observed using scanning electron microscopy (SEM) and transmission electron microscopy (TEM). Figure 1A showed that the self-assembled Au NPs array was closely packed and highly uniform in SERS platform. Compared to the 1-μm^2^ scale of the laser focal spot, a 10–100 folds scale of the self-assembled Au NPs arrays was large enough for the SERS detection, which is very important for the reproducibility of SERS detection. The average diameter of Au NPs was about 40 nm, see Figure 1B, and the interval between Au NPs was about 2−3 nm, see Figure 1C. These gaps between Au NPs can produce a strong electromagnetic enhancement (EM) that is beneficial for significant SERS enhancement. Therefore, the self-assembled Au NPs arrays can also ensure the sensitivity of SERS detection. The surface plasmon resonance (SPR) band of Au NP arrays on a quartz glass surface was about 660 nm, see Figure 1D, which were red-shifted by about 129 nm when compared to that of Au NP colloids at 531 nm, see Figure S1. The result indicated that strong surface plasmon resonance between adjacent Au NPs happened in a closely packed Au NPs array [34].

### 3.2. Colocalization of Au NPs and Pesticides Residues at Interfacial Array

**Figure 2:**
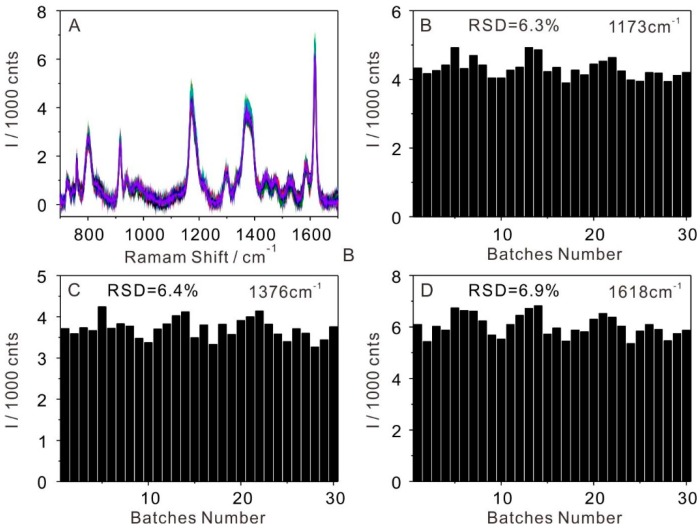
(**A**) A series of surface-enhanced Raman scattering (SERS) spectra of crystal violet (CV) molecules collected in 30 batches of self-assembly Au film and (**B**–**D**) histograms of SERS intensity of 30 spectra at 1173, 1376, and 1618 cm^−1^ in part A.

To analyze the colocalization of Au NPs and analytes in the array and possible fluctuations in every batch, SERS measurements of crystal violet (CV) were obtained in 30 batches of the self-assembled Au NP arrays after transferring it onto a silicon wafer, see Figure 2. Figure 2A showed 30 SERS spectra, each of which was obtained at a random point on 30 batches of self-assembly arrays. The Raman bands at 914, 805, and 726 cm^−1^ correspond to the symmetric stretch of the dimethylamino group, respectively. The band at 1173 cm^−1^ was assigned to the in-plane aromatic C–H bending modes. The Raman bands at 1535, 1584, and 1618 cm^−1^ were intense peaks of the main CV vibrational features of the carbon skeleton stretching modes. The spectra showed the symmetrical N–C-ring-C–C stretching mode of CV at 1352 and 1376 cm^−1^ [35,36]. The intensity of the main vibration of CV from 30 batches showed the excellent reproducibility of the interfacial self-assembly Au NP arrays. To get a statistically significant result, the relative standard deviations (RSD) of the band intensities at 1173, 1376, and 1618 cm^−1^ of the 30 SERS spectra were calculated in Figure 2B–D, respectively [37]. The RSD of the three Raman vibrations were 6.3%, 6.4%, and 6.9%, respectively, which clearly indicated the high reproducibility of the substrate.

### 3.3. Analysis of Paraoxon in Aqueous Phase by the Self-Assembly SERS Platform

**Figure 3:**
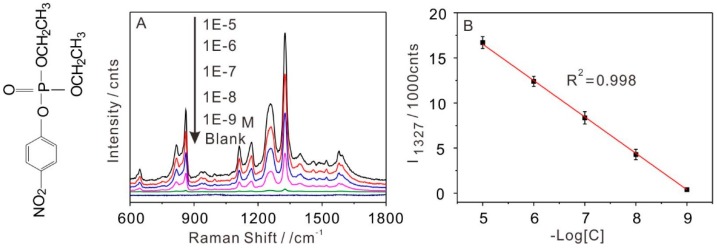
(**A**) SERS spectra obtained from different concentrations of paraoxon using self-assembly Au NPs arrays and blank substrate, the left of Figure 3A is the molecular structure of paraoxon; (**B**) the linear correlation of Raman intensity at 1327 cm^−1^ with the logarithm of paraoxon concentration.

To examine the sensitivity and practical application of the interfacial self-assembly array, the mixed aqueous solution of Au colloidal and paraoxon with different concentrations were used to self-assemble the SERS platform and directly detect paraoxon after transferring it onto silicon (Si) wafer. The SERS intensity of paraoxon increased as the paraoxon concentration increased from 10^−5^ to 10^−9^ mol/L (M) at the self-assembly Au NPs arrays, see Figure 3A. The SERS spectra from a bare Au NPs array was used as the background signal. All the SERS spectra showed the characteristic Raman bands of paraoxon molecules. The Raman band at 1258 and 1576 cm^−1^ correspond to the aromatic ring (C–O) and aromatic ring (C=C) stretching, respectively [38]. The spectra showed the symmetry stretching NO_2_ mode of paraoxon at 1327 cm^−1^. The band at 1110 cm^−1^ was assigned to the C–H band (in-plane)/NO_2_ asymmetric stretching. The band at 869 cm^−1^ was assigned to an aromatic–NO_2_ scissor; the band at 645 cm^−1^ corresponded to the C–C bending and aromatic NO_2_ shear mode [39,40].

The intensity of the strongest peak at 1327 cm^−1^, see Figure 3B, was used for the quantitative evaluation of the detection level and exhibited a good linear relationship with the concentration ranging from 10^−5^ to 10^−9^ M, and the correlation coefficient (R^2^) was 0.998. According to tests from three repeats, the limit of detection (LOD) of paraoxon was as low as 10^−9^ M by using the self-assembled Au NPs array. Based on the intensity of the symmetry stretching NO_2_ mode of paraoxon at 1327 cm^−1^, the EF of self-assembly Au NPs array was calculated to be 1.26 × 10^7^ according to the classical formula, see Supplementary Material Figure S2 [41], which showed good SERS enhancement of the substrate. The results above demonstrated that the different concentrations of paraoxon molecules can be loaded into Au NPs arrays and directly detected after transferring it onto a Si wafer.

### 3.4. Multiplex and Multiphase Detection of Pesticide Residue by the Self-Assembly SERS Platform

**Figure 4:**
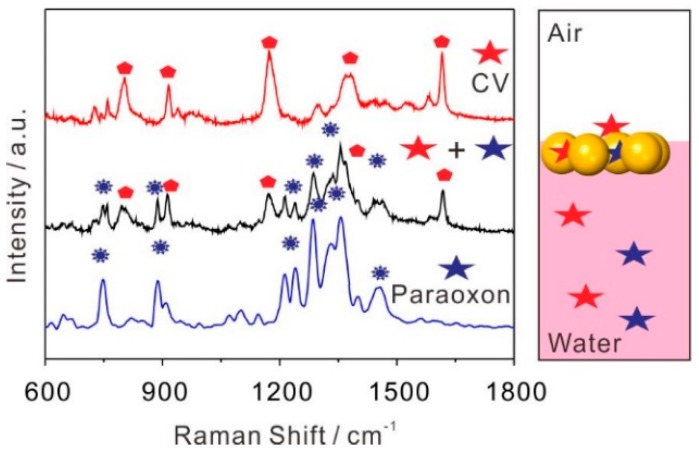
Self-assembly SERS platform for dual-analyte (black line) detection of 10^−6^ M paraoxon and 10^−6^ M CV in aqueous phase. SERS spectra of sole paraoxon (blue line) and CV (red line) in the aqueous phase, respectively, were measured as references.

We showed that the paraoxon molecule can be detected by the self-assembly Au NPs array easily without other interferents, see Figure 3. Here, we further inspected the detection performance of a self-assembly Au NPs array in the presence of other interferents. CV was introduced into the system for simultaneous detection. A mixed aqueous solution of colloidal Au with 10^−6^ M paraoxon and 10^−6^ M CV were used to self-assemble the SERS platform and two analytes were directly detected after transferring the substrate to Si wafer. The spectra of sole paraoxon and CV were used as references, see Figure 4. Figure 4 indicated that the information of paraoxon and CV could be detected together when comparing a dual-analyte SERS spectrum with those for sole analyte detection, see the black line in Figure 4. The peaks at 869, 1258, and 1327 cm^−1^ can be assigned to paraoxon, whereas the peaks at 806, 916, 1176, 1295, and 1618 cm^−1^ can be explicitly attributed to CV, exhibiting an excellent capability for dual-analyte detection by the self-assembly Au NPs array.

**Figure 5:**
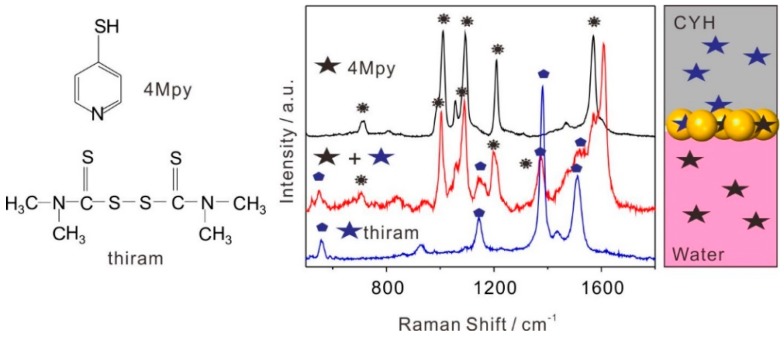
Self-assembly SERS platform for multiphase (red line) detection of 10^−6^ M thiram in organic phase and 10^−6^ M 4Mpy in aqueous phase. SERS spectra of sole thiram in organic phase (blue line) and sole 4Mpy in aqueous phase (black line), respectively, were measured as references. The left of Figure 5 was molecular structure of 4Mpy and thiram.

To test the general applicability of this method, we selected another pesticide, thiram as the analyte for detection. Meanwhile, we further examined the multiphase detection capability of self-assembly Au NPs arrays, such as aqueous and organic phases, see Figure 5. We added 4-mercaptopyridine (4Mpy) into the colloidal Au solution as the aqueous phase, and the final concentration of 4Mpy was 10^−6^ M. CYH with thiram (10^−6^ M) was used as the organic phase. These complex solutions were used to directly induce the self-assembly of Au NPs arrays and then two analytes were detected after transferring the substrate onto a Si wafer. The spectra of sole thiram and 4Mpy were also used as references. The red SERS spectrum in Figure 5 showed fingerprint Raman vibrations of both thiram and 4Mpy molecules when compared with those obtained from sole analyte detection. The peaks at 556, 1144, 1375, and 1506 cm^−1^ can be assigned to ν(S–S) band, ρ (HCN)+(CH_3_), δ_s_(CH_3_) coupled with ν(C–N) and δ_as_(CH_3_) coupled with υ(C–N) of thiram, respectively [42]. The peaks at 1010, 1093, 1209, and 1608 cm^−1^ can be explicitly indexed to 4Mpy [43,44]. These results confirm that the self-assembly SERS platform has high reliability, universality, and sensitivity to meet the requirements of pesticides residues detection under complex and unknown environments.

## 4. Conclusions

In summary, we showed a SERS platform with multiple hotspots by the self-assembly Au NP arrays for trace pesticides residues detection under complex environments. This SERS platform with A simple and rapid strategy can efficiently capture analytes in nanogaps during the interfacial arrays self-assemble process. Moreover, the interfacial self-assembly arrays can be easily transferred onto the silicon wafer, which can effectively avoid the interference of a background signal from a complex system. More importantly, the method is self-healing, easy to operate and handle when compared with traditional substrates. Lastly, our strategy has good sensitivity, reproducibility, and practicability for dual-analytes multiphase detection and can be extended to other small-molecular targets. We believe that this self-assembly SERS platform holds great promise for the rapid and trace detection in various complex fields, such as contaminated water, serum, and urine.

## Supplementary Materials

The following are available online at https://www.mdpi.com/2079-4991/9/3/426/s1, Figure S1: UV-vis absorbance spectra of Au NPs colloids; Figure S2: The enhancement factor (EF) of self-assembly Au NPs array, the SERS spectra of 10^−7^ M paraoxon solution (red line) and the normal Raman spectra of 0.1 M paraoxon solution (black line).
