# Supplementary material for: Surface-Enhanced Raman Spectroscopy on Self-Assembled Au Nanoparticles Arrays for Pesticides Residues Multiplex Detection under Complex Environment
Source: Nanomaterials (Basel). 2019 Mar 13;9(3):426. doi: 10.3390/nano9030426 (PMC6473963; doi:10.3390/nano9030426)
Supplement: Supplementary file 1 [file nanomaterials-09-00426-s001.pdf]

Supplementary section

Surface-Enhanced Raman Spectroscopy on Self-Assembled Au Nanoparticles Arrays for pesticides residues multiplex detection under complex environment

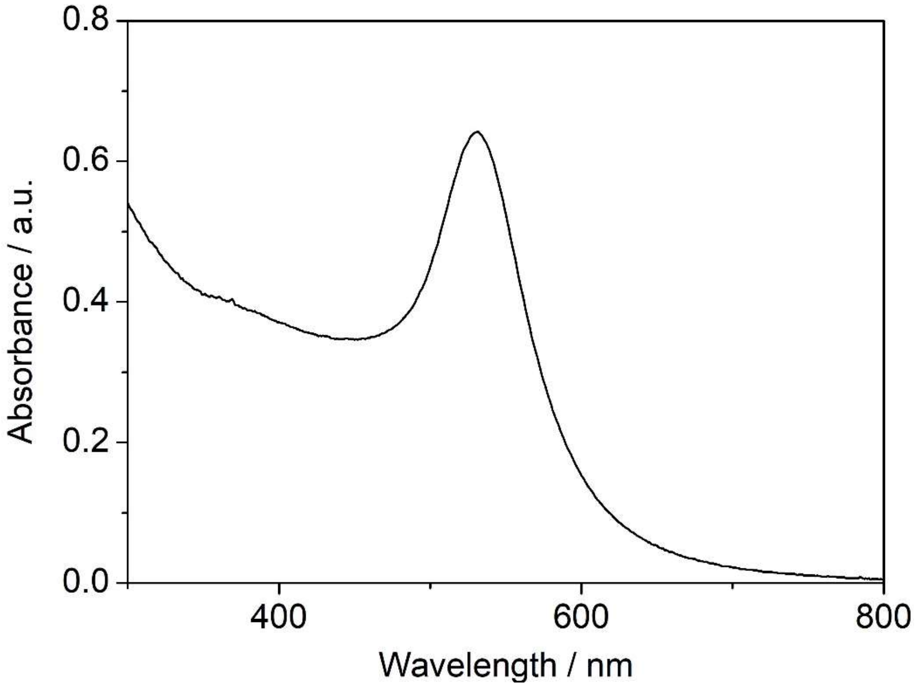

Fig. S1. UV-vis absorbance spectra of Au NPs colloids.

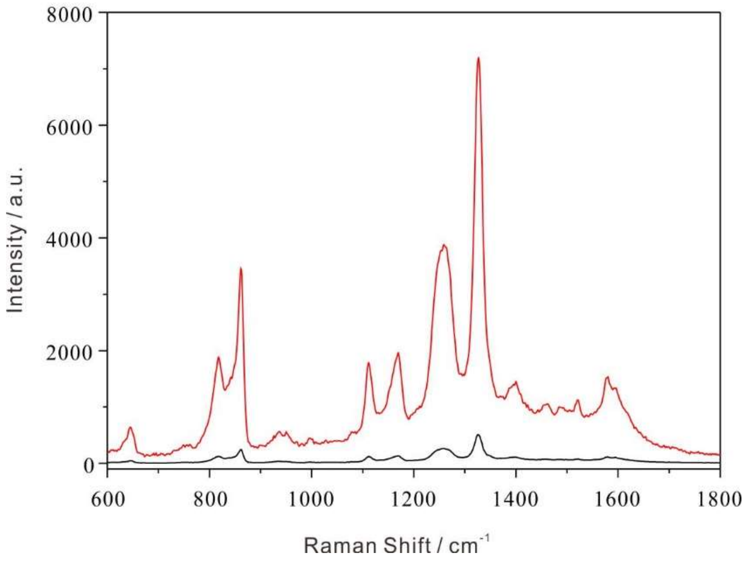

Fig. S2 The enhancement factor (EF) of self-assembly Au NPs array, the SERS spectra of 10^{-7} M paraoxon solution (red line) and the normal Raman spectra of 0.1 M paraoxon solution (black line).

For quantification, the enhancement factor (EF) was calculated using EF=$\frac{I\ SERS/_{N\ Surf}}{I\ RS/_{N\ vol}}$, or EF=$\frac{I\ SERS/_{C\ SERS}}{I\ RS/_{C\ RS}}$ when the number of molecules is below a single-molecule layer.[1, 2] Where *C RS* and *I RS* are the concentration and peak intensity for the regular Raman measurement with 0.1 M paraoxon solution on Si wafers respectively; and *C SERS* and *I SERS* are the concentration (10^{-7} M) and intensity for SERS measurement, respectively. Based on the intensity of the symmetry stretching NO_2 mode of paraoxon at 1327 cm^{-1}, the EF of self-assembly Au NPs array was calculate to be 1.26 $*10^7$ (EF=5982.629*0.1/(10^{-7}*474.153)=1.26*10^7) according to the classical formula, which showed a good SERS enhancement of the substrate.

1. E. C. Le Ru, E. Blackie, M. Meyer and P. G. Etchegoin, *Journal of Physical Chemistry C*, 2007, **111**, 13794-13803.
2. X. Zhou, F. Zhou, H. L. Liu, L. B. Yang and J. H. Liu, *Analyst*, 2013, **138**, 5832-5838.
